# Similar but different: distinct roles for KRAS and BRAF oncogenes in colorectal cancer development and therapy resistance

**DOI:** 10.18632/oncotarget.4750

**Published:** 2015-07-30

**Authors:** Markus Morkel, Pamela Riemer, Hendrik Bläker, Christine Sers

**Affiliations:** ^1^ Charité Universitätsmedizin Berlin, Institute of Pathology, Laboratory of Molecular Tumor Pathology and Systems Biology, Berlin 10117, Germany; ^2^ DKTK, German Consortium for Translational Cancer Research, Partner Site Berlin and DKFZ, German Cancer Research Center, D-63170 Heidelberg, Germany; ^3^ Charité Universitätsmedizin Berlin, Institute of Pathology, Berlin 10117, Germany

**Keywords:** KRAS, BRAF, colorectal cancer, signaling, therapy

## Abstract

Colorectal cancer (CRC) is characterized by recurrent mutations deregulating key cell signaling cascades and providing the cancer cells with novel functional traits. Among the most frequent mutations in CRC are gain-of-function missense mutations in *KRAS* and *BRAF*. Oncogenic activation of KRAS and BRAF is mutually exclusive and occurs in approximately 40% and 10% of all CRCs, respectively. Here we summarize genetic alterations currently described in the literature and databases, indicating overlapping but also specific co-occurrences with either mutated *BRAF* or *KRAS*. We describe common and potentially specific biological functions of KRAS and BRAF oncoproteins in the intestinal epithelial cells and during initiation and progression of CRC. We discuss signal transduction networks, highlighting individual functions of oncogenic KRAS and BRAF in terms of feedback loops and their impact on treatment outcome. Finally, we give an update on current strategies of targeted therapeutic intervention in oncogenic RAS-RAF signaling networks for the treatment of metastatic CRC and outline future directions.

## INTRODUCTION

### KRAS and BRAF as main players in the MAPK network

Cancer cells rely on signaling networks that are self-sufficient in providing growth signals and are refractory to growth inhibitory or apoptosis signals. This is due to multiple activating mutations in proto-oncogenes and functional loss of tumor suppressor genes [1]. KRAS and BRAF are major oncogenic drivers of colorectal cancer (CRC). They play further important roles in other cancer entities. Roles of KRAS and BRAF in other cancers are not discussed here, but have recently been reviewed elsewhere [2]. KRAS, a small GTPase, acts as a central relay for signals originating at receptor tyrosine kinases such as the EGFR family in the intestinal epithelium and in many other tissues [3]. Receptor tyrosine kinases stimulate KRAS activity via guanine nucleotide exchange factors, which activate KRAS by favoring GTP binding. The negative control is exerted through GTPase-activating proteins, which promote hydrolysis of GTP and thus KRAS inactivation. BRAF is a serine-threonine kinase that can be activated by KRAS and represents the top level element of the RAF-MEK-ERK (MAPK) kinase cascade [4]. MAPK signals regulate proliferation, differentiation, cell motility and further aspects of cellular activity via phosphorylation of many ERK substrates, such as cytoskeletal components and transcription factors. KRAS can also activate other signaling pathways in addition to the MAPK cascade. One of these is the PIK3CA-AKT-mTOR axis, which regulates protein translation and cell survival [5]. Together, the MAPK cascade and intersecting signaling pathways form a highly connected oncogenic network in CRC.

### Mutational patterns of *KRAS*, *BRAF* and other MAPK network genes in CRC

**Figure 1:**
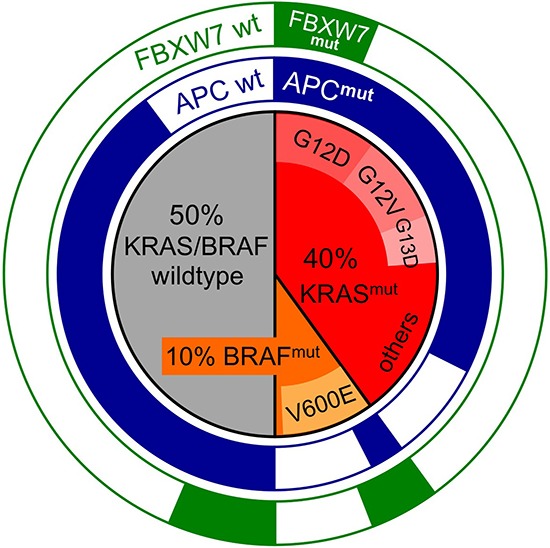
Mutational spectra of *KRAS*, *BRAF* and the Wnt effector genes *APC* and *FBXW7* in CRC Inner circle: fractions of KRAS-mutant (red) and BRAF-mutant (orange) and KRAS/BRAF-wildtype (grey) CRC. The most common mutations are given within the sections. Outer rings: relative proportions of APC-mutant (blue) or FBXW7-mutant (green) CRC found in KRAS-mutant, BRAF-mutant and KRAS/BRAF-wt CRC. APC mutations are significantly underrepresented in BRAF-mut CRC, while FBXW7 mutations are overrepresented. Mutational frequencies were derived from the TCGA and COSMIC databases.

Approximately 40% of CRCs display activating missense mutations in *KRAS* [6–8] (the COSMIC database reports 36% [9], while TCGA reports 42% of KRAS mutations [10, 11]; Fig. 1). These affect hotspots in codons 12 and 13 (80% of all KRAS mutations, of these are G12D > G12V > G13D > G12C > G12A), codon 61 (4% of all KRAS mutations, of these are Q61H > Q61L > Q61R) and 146 (1–2% of all KRAS mutations, mostly A146T and A146V). Furthermore, additional mutations in KRAS at various positions (e.g. 68, 117) are cataloged in the databases, yet their functional impact on KRAS protein function is largely unknown.

Structural analyses have presented a rationale for how the most frequent mutations activate KRAS: the glycine residues at positions 12 and 13 are important sites for interaction of KRAS with GAPs, while the glutamine at position 61 is a crucial site for the hydrolysis of GTP [12–14]. Therefore, mutations at either site lock KRAS in an active GTP-bound conformation constitutively presenting a docking surface for RAF kinases, including BRAF and CRAF (RAF1). Differences in clinical outcome have been identified in patients that harbor different KRAS mutations in the codons 12 and 13 [15, 16], although the data are discussed controversially and the mechanistic basis for these findings remains unknown. In addition to mutations, amplification of mutated *KRAS* and loss of heterozygosity resulting in elimination of the wildtype KRAS allele have been reported [11]. Since non-mutated RAS can limit the effects of oncogenic RAS, allelic imbalances favoring mutated *KRAS* could further supplement oncogenic KRAS signals [17].

*BRAF* mutations are less frequent in CRC [18] (COSMIC and TCGA report 11% and 10% of CRCs with activating mutations in *BRAF*; Fig. 1) [9–11]. *BRAF* mutations in CRC are mostly V600E amino acid substitutions, although various other mutations at codon 600 or neighboring positions within the kinase domain are documented, too. Structural studies of RAF proteins have identified the valine at position 600 as a crucial site within the conserved kinase domain, which is required for BRAF to maintain an inactive conformation in the absence of KRAS-BRAF interaction [19]. Mechanistically, mutations at this site likely render mutated BRAF independent from dimerization with BRAF or RAF1, which is normally a prerequisite for activation. Consequently, the V600E mutation is strongly activating, resulting in constitutive MEK binding, phosphorylation and therefore BRAF signal transduction. *BRAF* amplification and *BRAF* loss of heterozygosity have infrequently been detected in CRC [11]. The significance of these *BRAF* genomic imbalances is unclear, however *BRAF* copy number gains have been implicated in drug resistance of CRC [20, 21].

*KRAS* and *BRAF* mutations occur in a mutually exclusive manner in CRC [22] [9–11]. This may suggest that the mutations are functionally redundant during CRC development, i.e. no further selective advantage is provided for a cell by the second mutation when the first is already present. Another explanation for the mutual exclusivity is that mutations in KRAS and BRAF may be functionally incompatible; *BRAF* mutations would thus have unfavorable effects in KRAS-mutant CRC and vice versa, consequently leading to elimination of cells that have acquired both mutations sequentially. As a further explanation, *KRAS* or *BRAF* mutations could provide specific selective advantages that co-depend on the presence of other mutations. In support of this latter scenario, *APC* and *KRAS* mutations frequently co-occur, while APC and BRAF mutations show a significant trend towards mutual exclusivity [10, 11]. In contrast, mutations in the ubiquitin ligase *FBXW7* often co-occur with *BRAF* mutations, but are less frequent in *KRAS*-mutant or *KRAS/BRAF*-wildtype CRC (Fig. 1). This suggests that *KRAS*, but not *BRAF* mutations provide a selective advantage specifically in APC-mutant CRC precursor cells, whereas *FBXW7* mutations provide the greatest advantage for CRC cells harboring activated BRAF.

While mutations in *KRAS* and *BRAF* are the most frequent alterations in the MAPK cascade in CRC, further mutations involving other genes have been found. The *KRAS* homologue *NRAS* (but not the third RAS family member *HRAS*) harbors mutations in 2–4% of all CRCs, clustering at the amino acid residues Q61 and G12 [9, 11, 23]. It has been argued that the distinct mutational patterns within the RAS family are due to non-redundant regulatory mechanisms and individual cellular functions of KRAS, NRAS and HRAS [24, 25]. *ARAF* and *RAF1* do not show mutations in CRC. A probable explanation for the prevalence of *BRAF* mutations within the RAF gene family is the presence of a BRAF-specific domain that allows binding of additional interaction partners for RAS interaction [26, 27]. Alternatively, it may be due to the unique mode of activation of BRAF, which is primed for MEK phosphorylation [19, 28, 29]. Indeed, BRAF is a more potent signal transducer from RAS to the MEK-ERK kinases compared to ARAF or RAF1 [30, 31]. In addition to RAS and RAF family members also *NF1*, coding for the RAS-regulatory GTPase-activating protein Neurofibromin 1, has been found mutated in some (approx. 4%) CRCs [32]. Recently, it has been shown that inactivation of *NF1* can synergize with oncogenic KRAS and also potentially with non-canonical KRAS mutations [33]. Furthermore, another RAS-GAP encoding gene, *DAB2IP*, displays mutations in approximately 8% of all CRCs, as reported by TCGA. While the nature and consequences of these non-synonymous SNPs is currently unknown, the observations point towards more prominent roles of RAS-associated regulatory processes than previously anticipated. A potential tumor-suppressive role of DAB2IP has recently been highlighted in a prostate cancer model, where *DAB2IP* gene loss activated both RAS and NFκB [34]. In addition, high DNA methylation frequencies of the *DAB2IP* gene have been found in multiple human cancers [35]. Mutations with functional impact on MAPK signal transduction in genes encoding the more downstream MEK and ERK kinases have not been reported in CRC. However, approximately 6% of CRCs harbor alterations within the *MAP2K4* gene, also called JNKK, a serine threonine kinase within the stress-activated MAPK pathway [10]. Together, alterations in *KRAS, NRAS*, *BRAF, NF1, DAB2IP* and *MAP2K4* comprise 64% of the CRC tumors presented by the TCGA dataset. Only *BRAF* and *KRAS* mutations appear significantly mutually exclusive, the other mutations can occur within the same samples.

### *KRAS* and *BRAF* mutations occur in distinct sequences and patterns during CRC development

Activation of the EGFR-RAS-RAF and the Wnt-APC-β-Catenin signaling axes represent key steps in initiation and early progression of CRC [36, 37]. Indeed, EGFR signals, together with Wnt and Notch signals, form part of a larger signaling network controlling the maintenance of stem cells and the proliferative compartment of the normal intestinal epithelium [38–41]. Pathway-activating mutations represent essential steps during the early phases of CRC development, because they favor stem cell and proliferative characteristics independently of ligands provided by the microenvironment [42–44].

**Figure 2:**
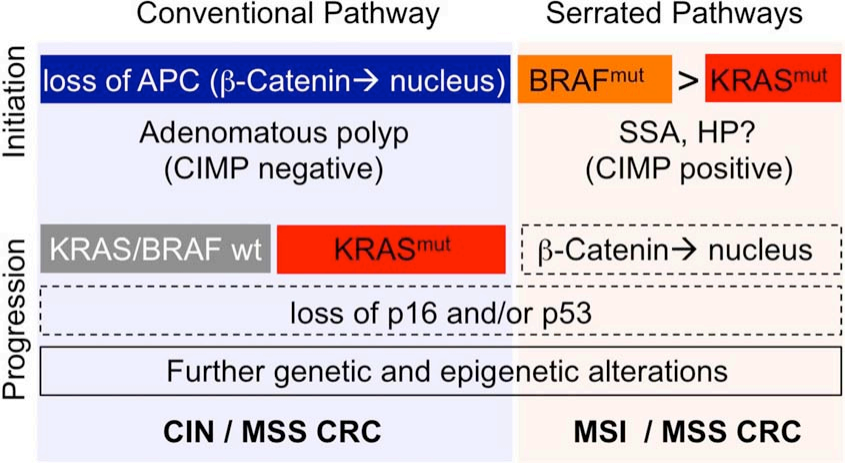
Roles of KRAS (red) and BRAF (orange) in distinct progression pathways of CRC A majority (70–80%) of CRCs initiate via APC mutations (blue) and develop via conventional adenomatous polyps (to the left) that show no or low methylation of CpG islands (CIMP-negative/low). KRAS, but not BRAF, mutations are frequent in this group. CRC developing via this route are associated with microsatellite-stability (MSS) and chromosomal instablity (CIN). A minority (20–30%) of CRCs initiates via BRAF or KRAS mutations and develop via serrated adenoma precursors (to the right). Sessile-serrated adenoma (SSA) is highly associated with BRAF mutations. The malignant potential of hyperplastic polyps (HP) is not well defined. BRAF-mutant SSA frequently shows nuclear β-Catenin, but APC mutations are rare. Serrated tumors, in particular these with BRAF mutations, often show strong methylation of CpG islands (CIMP-high). CRC forming via the BRAF/SSA pathway are microsatellite-stable or microsatellite-instable (MSS/MSI). It is of note that the diagram summarizes only major correlations between molecular and clinical features. APC, BRAF and KRAS mutations are given in blue, yellow and red. Further genetic and epigenetic alterations arising during tumor progression are outlined. Dashed boxes indicate events that are frequent but not mandatory in the progression pathway. Precursor lesions and prevalent methylation phenotypes are given below the initiating mutations. The most common molecular CRC types are given in bold, below the progression pathways.

The EGFR-RAS-RAF and the Wnt-APC-β-Catenin signaling cascades can be activated by different mutations and in different order (Fig. 2). There is ample evidence that different mutational sequences can give rise to different forms of precancerous (adenomatous) lesions. A majority (approx. 70–80%) of CRCs develop via conventional adenomatous polyps that are initiated by mutations activating the Wnt/β-catenin pathway, commonly in *APC* [9, 11, 45]. Analyses of different stages of human neoplasia and analyses of differently-sized adenomas revealed that *KRAS* mutations are rare (10%) in small and early adenomas but frequent (50%) in larger and more advanced adenomas [8, 46]. These data imply that *KRAS* mutations often occur after the initiating *APC* mutation in CRC developing from adenomatous polyps. Indeed, mouse tumor models have shown that oncogenic KRAS synergizes with loss of APC in intestinal tumor progression: while APC inactivation results in the formation of benign adenoma only, activation of oncogenic KRAS in combination with APC inactivation results in the growth of invasive adenocarcinomas [47, 48]. One mechanism contributing to synergistic effects of the mutations is the convergence of KRAS-RAF1 and APC activities to promote nuclear localization of β-Catenin and subsequent activation of intestinal cell proliferation [49]. The sequence of events with *APC/Wnt* being the initiating mutation and *KRAS* a tumor promoting mutation is probably unique to *APC* and *KRAS*. To the best of our knowledge, it has not been demonstrated that *BRAF* mutations can follow *APC* mutations during the development of conventional adenoma, suggesting that BRAF does not perform strictly equivalent functions as KRAS in the intestinal epithelium to promote transformation and cancer progression.

A minor proportion (approx. 20–30%) of CRCs develop via serrated precursors, such as sessile serrated adenoma or hyperplastic polyps [50–52]. There are multiple lines of evidence that serrated adenomas are initiated via activating mutations in the EGFR-RAS-RAF signaling axis without prior APC inactivation. A study concentrating on aberrant crypt foci, representing the earliest precursors of CRC, found 10 out of 16 (63%) serrated foci to display *BRAF* mutations, but only 1 of 33 non-serrated crypt focus was *BRAF*-mutant [53]. Another study examined intestinal adenoma, and found *BRAF* mutations in all (9/9) dysplastic serrated adenomas and also in 18/50 (36%) hyperplastic polyps characterized by elongated crypts [54]. In contrast to *BRAF* mutations, *KRAS* mutations were comparatively infrequent in serrated crypt foci (3/16), absent in dysplastic serrated adenoma (0/9), but present in 9/50 hyperplastic polyps [53, 54]. Recent studies confirmed and extended these results using genomic profiling [55]. In line with the data from human precancerous lesions, several mouse models of activated MAPK signaling display serrated intestinal hyperplasia or dysplasia: overexpression of EGF ligand results in serrated polyp formation in the cecum [56], and inducible knock-in *KRAS(G12D)* mice develop serrated hyperplasia of the small and large intestine [57–59]. Likewise, activation of oncogenic BRAF in the intestine results in formation of generalized serrated hyperplasia [60, 61]. Analysis of *BRAF*-mutant foci progressing via dysplastic stages to low- and high-grade carcinoma revealed a preferred sequence of events in mouse serrated tumor progression [61]: following the initiating *BRAF* mutation, MAPK activity was only mildly enhanced. Advanced dysplastic foci contained further mutations that activated the Wnt/β-catenin pathway, such as activating mutations in *Ctnnb1* (coding for β-Catenin), or inactivating mutations in the negative Wnt regulators *Apc* or *Lrp1b*. These together with the initiating *BRAF* mutation and possibly further genomic alterations resulted in a hyperactivation of both Wnt and MAPK signals. This progression sequence for murine serrated adenoma is in line with data from human sessile serrated adenoma, which also displays both *BRAF* mutations and nuclear β-Catenin [62, 63].

Several key lessons can be learned from the collective studies of human CRC progression and mouse models. Firstly, oncogenic *KRAS* is a key factor in the progression of conventional adenoma by synergizing with the initiating functional loss of *APC*. Secondly, both oncogenic *BRAF* and *KRAS* can act as initiators in the development of CRCs arising via serrated adenoma. However, since *BRAF* mutations are strongly associated with sessile serrated phenotypes, their individual effects as tumor initiators in the intestinal epithelium are clearly different. Thirdly, the observation of nuclear β-catenin in serrated adenomas that arise via *BRAF* mutations strongly suggests that high Wnt/β-catenin activity is favored at an early stage of serrated tumor progression via mechanisms yet unknown in human CRC. The TCGA database provides a rich source of such alterations (such as SNPs, gene fusions and copy number alterations), and reveals that *BRAF*-mutated tumors are associated with multiple mutations in Wnt pathway-associated genes such as *AXIN1, CDX2, SOX9, FBXW7, TCF7L1/2* and *RSPO1/2* [64]. The co-occurrence between mutations in *BRAF* and *FBXW7* is significant *( p*-value < 0.001; survey TCGA, May 2015), indicating that mutated *BRAF* preferentially co-operates with distinct Wnt signal modulators (Fig. 1). In contrast to conventional adenoma, mutations in *APC* are an exception rather than the rule in the developmental pathway of serrated adenoma driven by BRAF [65].

A further important observation from mouse models and human CRC is that mutations activating KRAS and BRAF do not necessarily result in tumor formation in the intestinal epithelium, due to the existence of fail-safe mechanisms suppressing tumor growth after MAPK activation. In humans, this is evidenced by the existence of many serrated aberrant crypt foci, which are dormant and do not progress [53]. In mice, oncogenic activation via knock-in *KRAS* and *BRAF* alleles has likewise been associated with the induction of oncogene-induced senescence in some [59, 60], but not all [48, 58, 61, 66] models, suggesting that the response to oncogenic MAPK signals is highly context-dependent. In oncogenic *KRAS* as well as in *BRAF* mouse models, cells could escape senescence following the deletion of p16INK4a or p53 [59, 60], and these mutations are also frequent in human CRC. It thus appears that tumor initiation and/or progression by *KRAS* or *BRAF* not only depends on mutational activation in the tumor-initiating cell, but also on successful evasion of common tumor suppressing mechanisms in the mutated clone.

### KRAS and BRAF differentially regulate cellular hierarchies, stem cell function and CRC development

Recent studies in mice have unveiled distinct effects of *KRAS* and *BRAF* oncogenes on cellular hierarchies in the normal intestinal epithelium. For instance, both oncogenic KRAS and BRAF can direct differentiation towards secretory Goblet cells in the mouse intestine [58, 61, 67]. Furthermore, both oncogenes were found to affect stem cell fate: using clonal analyses, two studies have noted that the progeny of intestinal stem cells expressing oncogenic *KRAS* expands within and beyond single crypts by modulating asymmetric stem cell division [68, 69]. It is of note that the stem cell pool does not necessarily expand while the *KRAS* mutation spreads in the tissue, due to further and poorly defined mechanisms sustaining stem cell homeostasis at the crypt level [67]. Furthermore, the formation of ectopic stem cells in the differentiated villus tissue has been observed after activation of *KRAS(G12D)* in the intestine of mice [70]. In contrast to this, we found that generalized transgenic expression of oncogenic *BRAF* in the intestine results in a rapid depletion of the entire stem cell pool, which adopts progenitor fate [71]. A reduction of intestinal stem cell markers is also found in hyperplastic intestinal tissue of *BRAF(V637E)* knock-in mice [61]. It therefore appears that the *KRAS* and the *BRAF* oncogenes modulate signaling networks controlling homeostasis and stem cell competition in the intestinal crypt in an opposing manner. Side-by-side studies using comparable genetically engineered mouse models will be required to ascertain these effects, which could shed light on different selective constraints that KRAS- versus BRAF- mutant cells have for tumor initiation and progression in the intestinal epithelium.

In agreement with the possibility of differential functional roles of oncogenic *KRAS* and *BRAF* mutations during early stages of tumor development, significant correlations with molecular and clinical features have been identified (Fig. 2). In particular, CRCs with mutant *BRAF* are characterized by specific genetic and epigenetic features [11, 65, 72–75]. On the level of the epigenome, *BRAF*-mutant CRCs and their (serrated) precursors often display genome-wide hypermethylation of CpG islands (*C*pG *I*sland *M*ethylator *P*henotype: CIMP-high). A recent study suggests that the association between mutant *BRAF* and CIMP-high is due to the phosphorylation of the transcriptional co-repressor MAFG via the BRAF-MEK-ERK axis. Subsequently, complexes of phosphorylated MAFG, BACH1 and the epigenetic modifiers CHD8 and DNMT3B are recruited to CpG islands, resulting in focal DNA hypermethylation and transcriptional silencing of nearby genes [76]. *BRAF*-mutant CRCs are also characterized by mismatch-repair deficiency, high levels of microsatellite instability (MSI-H) and very high overall mutation rates (>12 mutations per 10^6^ nucleotides) [22]. Clinically, *BRAF* mutations are associated with infiltration of lymphocytes, localization in the proximal/right colon, occurrence in female patients, poor differentiation, mucinous (i.e. Goblet-cell rich) type. The association with these features is well explained by the overlap of *BRAF* mutations with microsatellite instability (MSI-H), a common finding in the serrated pathway of colorectal cancer. *BRAF* mutations have also been associated with poor survival, but this phenomenon is restricted to carcinomas not showing microsatellite instability [75]. In contrast, *KRAS* mutations are more frequent in CRCs of the left colon and in male patients. *KRAS* mutations are associated with microsatellite-stability (MSS) or low levels of microsatellite-instability (MSI-L), and lower rates of gene methylation (CIMP-negative or low). Frequencies of *BRAF* and *KRAS* mutations, as well as rates of CIMP and MSI form continuous gradients along the longitudinal axis of the gut [77, 78]. This suggests that CRC does not emerge as distinct subtypes that occur strictly in the different parts of the colon and rectum. Indeed, rectal cancer matches colon cancer regarding most of its molecular features [11], but has a lower incidence of *BRAF* mutations and patients may have individual clinical requirements [79, 80]. More recently, integrated unsupervised analyses of genetic and epigenetic traits have supported the idea that distinct classes of CRCs exist along borders defined by gene expression, genome stability (such as MSI/MSS), epigenetic make-up (such as-CIMP high/low/negative) and *BRAF/KRAS* mutational status [11, 81–83]. In these classifications, CRCs with *KRAS* or *BRAF* mutations are regularly enriched in different classes, highlighting their different evolutionary histories and distinct wiring of signaling networks.

### Similar and distinct roles of BRAF and KRAS in signal transduction and therapeutic intervention

**Figure 3:**
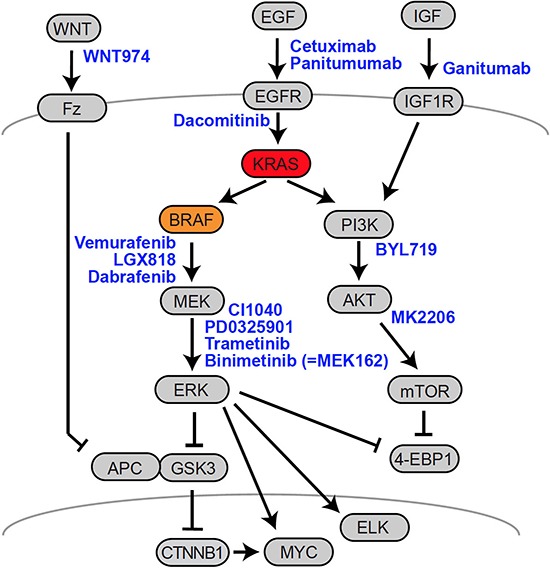
Therapeutic targets in CRC A schematic representation of the EGFR-RAS-MAPK, PI3K and Wnt-APC-β-Catenin signaling axes is given, along with therapeutics used in clinical studies. Arrows indicate important connections between the signaling molecules. Drugs are given in blue, next to their targets. Names of proteins frequently refer to a representative member of a multiprotein family. For further details, see main text. For a list of clinical studies, see Table 1.

During tumor progression, genetic (and epigenetic) alterations accumulate in an evolutionary manner via consecutive cycles of mutation and selection. Multiple mutations ultimately contribute to the formation of an oncogenic network sustaining the transformed cancer phenotype. In the oncogenic signal networks of advanced CRC, mutated KRAS and BRAF have been shown to serve many functions beyond maintaining cellular proliferation, stemness and growth factor-independent growth. Indeed, both oncoproteins have been shown to contribute to angiogenesis, cell differentiation, epithelial-mesenchymal transition, adaptations of cellular metabolism and circadian rhythm networks, and many further traits of tumor cells. [3, 4, 84–87]. Due to the complex patterns of interdependent driver mutations, cancer cells frequently become dependent on certain oncogenic signals such as for oncogenic *KRAS* in CRC [88]. This phenomenon—designated as “oncogene addiction”—opens a therapeutic window for the specific targeting of cancer [89, 90]. The essential role of the hyperactivated EGFR-KRAS-BRAF signaling cascade in CRC has spurred the development of therapeutic approaches to inhibit the cascade on several levels, specifically targeting EGFR, KRAS and BRAF (Fig. 3, Table 1). KRAS itself, being a small GTPase, has proven to be largely “undruggable” to date, despite recent promising developments [91].

**Table 1:** Clinical studies employing targeted combination therapies for the treatment of advanced CRC with specific mutation patterns

Study Identifier	Condition (Mutations)	Intervention	Primary Outcome	Phase
NCT01791309	BRAF(V600E) CRC	Vemurafenib Panitumumab	ORR	Pilot
NCT01719380	BRAF-mut CRC	LGX818 Cetuximab BYL719	Toxicity, PFS	I/II
NCT01750918	BRAF(V600E) CRC	Dabrafenib Trametinib Panitumumab 5-fluorouracil	Safety, RR, PFS	I/II
NCT02278133	BRAF-mut CRC + upstream Wnt pathway activation	WNT974 LGX818 Cetuximab	Toxicity, ORR	Ib/II
NCT01562899	KRAS-mut CRC	MEK162 Ganitumab	Toxicity, ORR	Ib/II
NCT02039336	KRAS-mut CRC	Dacomitinib PD0325901	Toxicity, PFS	I/II
NCT02399943	RAS/RAF-wt CRC	Panitumumab Trametinib	Response	II

For detailed information, refer to http://www.clinicaltrials.gov. For therapeutic targets, see Fig. 3. CRC = colorectal cancer; ORR = overall response rate; PFS = progression-free survival; RR = response rate.

Inhibition of the transmembrane tyrosine kinase receptor EGFR has proven to be beneficial for a considerable subset of patients with metastatic CRC. Upon treatment with EGFR-inhibiting antibodies such as Cetuximab or Panitumumab, patients showed an overall survival benefit of 3–5 months when the cancer was wildtype for *KRAS*, but no benefit when the cancer was *KRAS*-mutated [92–94]. Therefore, *KRAS*, and now also *NRAS* mutations are considered negative predictive markers for anti-EGFR therapy. Presently, Cetuximab and Panitumumab are recommended as first-line therapy in combination with chemotherapy for patients with wildtype configurations in *KRAS* and *NRAS* according to European (ESMO) and American (AJCC) standards. Other targeted therapies currently available in clinical routine, such as the VEGF inhibitor Bevazicumab, seem to act independently of both KRAS and BRAF [95].

In contrast to the *RAS* mutations, mutant *BRAF* has not been identified as an independent predictive marker for first-line anti-EGFR therapy in a dedicated clinical study. This is most likely due to the fact that *BRAF* mutations occur at rather low frequencies and thus no clinical study harbors enough patients to reach statistical significance. Furthermore, patients with *BRAF* mutations, in particular these with *BRAF/MSS*, have a poor outcome, which is independent of the applied therapy [75] [95]. However, a recent meta-analysis investigating the outcome of more than 400 *RASwt/BRAFmut* patients from 10 different trials clearly showed that patients harboring *BRAF* mutations do not benefit from EGFR-directed therapy and thus should be tested prior to the administration of either Cetuximab or Panitumumab [96].

It is important to note that even responders to anti-EGFR therapy routinely develop secondary resistance during anti-EGFR therapy, often by selection of *KRAS/NRAS* or *BRAF*-mutant clones arising from a RAS-wildtype cancer [97–99]. Indeed, mathematical modeling has suggested that targeted monotherapy will invariably lead to the selection of resistant cells once a cancer has grown beyond a certain size [100]. Interestingly, a recent CRC progression model suggested that clonal evolution of CRC is constrained by the organization of the cancer into distinct glandular crypts, effectively reserving evolutionary sweeps in the advanced disease to rare events such as metastasis and the emergence of therapy resistance [101, 102]. Therefore, anti-EGFR therapy could aid the expansion of KRAS- or BRAF-mutant clones that would be contained in a local niche in the absence of therapy.

It appears to be a rational strategy to target mitogenic signaling downstream of mutated *KRAS* and *BRAF*, since both mutations are prevalent in primary and resistant CRC and the mutations have a negative predictive and prognostic value. However, inhibition of oncogenic *BRAF(V600E)* using Vemurafenib, or of the MEK kinase using CI1040, has proven to be ineffective in CRC [103, 104]. The major reason for this disappointing outcome of kinase inhibition within the MAPK kinase cascade is the existence of multiple levels of feedback control, and regulatory intersections with further pathways such as PI3K-AKT.

**Figure 4:**
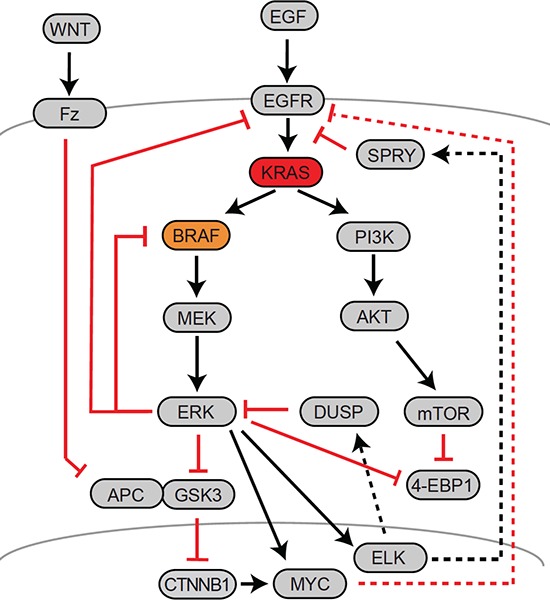
Major feedback mechanisms controlling MAPK activity in CRC A schematic representation of the EGFR-RAS-MAPK, PI3K and Wnt-APC-β-Catenin signaling axes is given, along with signaling connections. Major positive interactions are given as black arrows, while inhibitory interactions are given as red blocked lines. Solid lines indicate molecular interactions, whereas dotted lines indicate transcriptional control. Names frequently refer to a representative member of a multiprotein family.

Indeed, several levels of feedback exist between the RAF-MEK-ERK axis and upstream receptor tyrosine kinases such as EGFR (Fig. 4): firstly, active ERK phosphorylates EGFR and EGFR signaling adaptor molecules at inhibitory residues, thus keeping signal transduction of the ligand-bound receptor to RAS in check [105–107]. Secondly, ERK activity induces transcription of SPRY family feedback inhibitors that likewise control signal transduction from receptors to RAS proteins [108]. These levels of feedback limit growth factor-induced MAPK activation in the normal tissue. In CRC cells with constitutive MAPK activity, feedbacks converging on receptor tyrosine kinases are inactivated once MEK is inhibited. Thus, receptors including EGFR become activated under MEK blockade, triggering multiple downstream pathways such as MAPK and the PIK3CA-AKT-mTOR signaling axis (Fig. 4). In addition it has been found that the EGFR family member gene ERBB3 is upregulated by the transcription factor c-Myc upon MEK inhibition, suggesting further feedback control on the transcriptional level [109]. In line with negative transcriptional and posttranslational control of EGFR signals via ERK, recent siRNA screens and systems biology approaches have revealed that EGFR signal re-activation plays an important role in resistance to MEK blockade in CRC cells [110, 111]. In addition, ERK activity controls MAPK signals via mechanisms that provide feedback downstream of RAS. This level of control is exerted directly via inhibitory phosphorylation of RAF-1 by ERK [112], and also via transcriptional activation the DUSP family of phosphatases that dephosphorylate and therefore inactivate ERK [113]. Of the various layers of feedback control, the prevalent mechanism in CRC cells appears to be strong negative feedback from ERK1/2 to RAF [114]. Feedback activation of EGFR and RAF have also been identified as important mechanisms to mediate drug resistance following RAF or MEK inhibition [99, 110, 115–117].

The disappointing results achieved using the BRAF(V600E)-specific inhibitor Vemurafenib in CRC patients not only showed the importance of these feedbacks *in vivo*, but also demonstrated the different wiring of oncogenic networks in cancers of either neuroectodermal or epithelial origin such as melanoma and CRC, respectively. This led to the development of preclinical treatment schemes that appear counter-intuitive, but take into account the feedback-controlled organization of oncogenic networks that can only be controlled by simultaneous treatment with multiple drugs. Preclinical studies suggested improved antitumor activity when BRAF inhibition was employed in combinatorial treatment [118]. Currently, several pilot trials and clinical studies aim at simultaneously inhibiting EGFR and BRAF or MEK for treatment of BRAF-mutant (or KRAS-mutant) CRC patients, in order to block both, the oncogenic RAF-MEK-ERK signal, as well as the feedback loop via EGFR family members (Fig. 3, Table 1). First results suggest that a limited clinical response exists in patients that have failed in first-line therapies [119–124]. However, patients who received EGFR-MEK-BRAF combination therapies can also relapse with novel genetic alterations conferring resistance through sustained MAPK activity, such as amplification of KRAS or BRAF or, in one case, a mutation in the downstream kinase MEK1 [125]. It is indeed expected that combination therapies converging on a common (downstream) signaling pathway are prone to the development of therapy resistance [100].

It is important to note that KRAS- versus BRAF-mutant CRCs likely display characteristic differences in response to therapeutic interference in the MAPK cascade, due to mechanistic differences in signal transduction (Fig. 4). For one, BRAF mutations disallow the critical feedback from ERK to RAF to occur, while KRAS mutations leave this feedback intact [114]. As a consequence, higher levels of MEK inhibitor are required in KRAS mutated CRC cells as compared to BRAF mutated cells to suppress MEK/ERK activation. Furthermore, highlighting an important difference between KRAS- and BRAF-mutant cancer cells, ATP-competitive RAF inhibitors were found to block MEK-ERK signal transduction in BRAF-mutant cancer cells, but unexpectedly activated MEK-ERK signaling in cancer cells harboring mutant RAS and wildtype BRAF [126–128]. On a molecular level, this paradoxical activation could be explained by different propensities of BRAF and RAF-1 to form homo- versus heterodimers [4, 129]. It was also found that inhibition of active MEK has different constraints downstream of oncogenic KRAS versus BRAF [130]: while KRAS-driven cancer cells are sensitive towards inhibitors interacting with MEK-Serine212 (a site critical for feedback between MEK and wildtype BRAF), BRAF-mutant cancer cells required another class of MEK inhibitor that blocks phosphorylated active MEK. Taken together, the aforementioned studies underline the necessity to develop specific and effective diagnostics and therapies for patients with *BRAF* mutated CRC. An unusual approach to exploit specific traits of BRAF-mutated cells was recently presented by exploiting the finding that synthesis of BRAF is dependent on chaperone action. Thus, interference with the BRAF chaperone TRAP1 was shown to effectively inhibit proliferation of *BRAF* mutated CRC cells [131].

Finally, it is necessary to expand the view beyond the EGFR-RAS-RAF-MEK-ERK cascade to appreciate interactions within the larger signaling network and cover specific signaling properties of the oncogenes. As outlined above, feedback via EGFR is suited to activate PIK3CA-AKT signals upon MAPK blockade. In this regard, it is important that the PIK3CA-AKT-mTOR axis is frequently activated either by loss of PTEN or activation of PIK3CA in CRC, independent of the KRAS or BRAF status [132]. Furthermore, the RAF-MEK-ERK and PIK3CA-AKT-mTOR cascades converge on important common substrates, such as 4EBP1 and eIF4F, controlling protein translation, cell survival and ultimately therapy resistance [133, 134]. Therefore, novel combination therapies currently in clinical trials seek to inhibit the EGFR-RAS-RAF-MEK-ERK and PIK3CA-AKT cascades in parallel, for instance by combining Cetuximab, LGX818 and the PI3K inhibitor BYL719 in patients with BRAF mutations [135] (Fig. 3, Table 1). These strategies seem to be promising, given that both oncogenic pathways have common targets that only respond to the parallel inhibition of RAS-RAF-MEK and PIK3CA-AKT [133]. Indeed, concurrent inhibition of BRAF and PIK3CA/mTOR induced tumor regression in a BRAF-mutant CRC mouse model [136]. Adding another twist to the story, mTOR-4EBP1 signals are also controlled via the Wnt-APC-β-Catenin axis in the intestinal epithelium [137], providing yet another important convergence point of key signaling pathways. Simultaneous inhibition of Wnt and MAPK signals is currently in clinical testing for the treatment of a subset of BRAF-mutant CRC patients, employing Cetuximab, LGX818 and the Wnt ligand maturation inhibitor WNT974 [138](Fig. 3, Table 1). Recent preclinical studies suggest a role for the Hippo signal transducer YAP in resistance to RAF and MEK inhibition in multiple cancer cell lines, including BRAF-mutant CRC. These findings may provide another strategy for the design of combinatorial targeted therapies [139, 140].

Future models of tumor development and therapeutic intervention may also need to integrate the activities of soluble factors providing autocrine or paracrine interactions between different cells types that compose the tumor. Already in 2001, Schulze *et al*. described a positive autocrine feedback loop induced by RAF via the EGFR ligand HB-EGF [141]. KRAS-mutant CRC cells can also activate a positive loop via TGF-alpha, one of the strongest EGFR activators, which in turn results in the activation of a CXCL1/CXCL8-dependent autocrine signal [142]. Paracrine interactions between tumor and stromal cells have also been identified. RAF inhibitors were found to elicit innate resistance via secretion of the growth factor HGF by stromal cells, resulting in ERK and PI3K-AKT activation and therapy resistance [143]; these results suggest a potential for the concurrent application of RAF and HGF/MET inhibitors in CRC and other cancers. Furthermore, a major part of the gene expression differences between good- and poor-prognosis CRC were found to arise from TGF-β signals in cancer-associated fibroblasts [144]. Importantly, the blockade of TGF-β signals stopped CRC progression in *in-vitro* and xenograft models. Further expanding the idea of cellular and clonal co-operation in cancer, recent data from breast cancer suggests the existence of tumor cell subclones that can cooperate with stromal cells to support cancer growth via secreted factors [145]. While no comprehensive data on secreted factors determined by KRAS or BRAF oncogenes are available in CRC, this might comprise a further molecular level of specificity determining both the distinct roles of KRAS and BRAF in tumor progression and therapy response.

A recent study reported improved antitumor response in a mouse model of melanoma using BRAF and MEK inhibitors in combination with immunotherapy [146]. Such improved therapeutic applications are currently not available for *KRAS-* and *BRAF-*mutated CRC, however we have previously described a reversible negative impact of the KRAS oncogene and activated MAPK signaling onto MHC-I expression in CRC cells [147]. A recent publication confirmed a significant association between MHC-I loss and *KRAS* mutations, but not *BRAF* mutations, in CRC [148]. Consequently, similar to the observations in melanoma, the development of combinatorial treatment using BRAF and/or MEK inhibitors with immunotherapy appears to hold promise for CRC patients. However, an improved knowledge of underlying mechanisms and the plasticity of the contributing factors upon therapeutic interference is required.

### Outlook

Since the discovery of mutated forms of KRAS and BRAF as mutually exclusive drivers of colorectal carcinogenesis, much progress has been made in understanding their common and individual effects. Inhibition of oncogenic signals originating from KRAS and BRAF has a high priority in CRC research. However, inhibition of single nodes within the intracellular kinase networks associated with KRAS and BRAF typically resulted in drug resistance and therapy failure. Studies of human cancer specimens, cells and mice suggest that resistance to inhibitors is usually due to feedback and crosstalk mechanisms that allow transduction of oncogenic signals in spite of blockade of a single network node. In addition, inadvertent selection of resistant clones with novel combinations of oncogenic mutations can occur under monotherapy or combination therapy that allows for single mutation to confer drug resistance. Therefore, a better understanding of the BRAF and KRAS-specific wiring of the MAPK signaling network is required for the development of new combinatorial therapeutic options.

Yet the impact that oncogenic KRAS and BRAF have on CRC development stretches out beyond their roles as signaling molecules. It can be inferred from their similar but subtly different roles during tumor initiation that KRAS and BRAF are also defining factors influencing cancer development. This probably occurs via modulation of signaling networks impinging on the epigenome and mutation-specific constraints of signaling networks that dictate clonal selection. It will therefore be important to differentiate CRCs beyond the mutational status of BRAF and KRAS by further defining selective advantages and limitations of KRAS- and BRAF-mutant cells in a tissue context in order to understand their clonal histories and co-selected vulnerabilities. Thus, analysis of tumor evolution in combination with a better understanding of oncogenic signaling networks will provide cues to derive novel predictive markers and therapy options.
